# Glutaminase Reprogramming in Hepatocellular Carcinoma: Implications for Diagnosis, Prognosis, and Potential as a Novel Therapeutic Target

**DOI:** 10.3390/ijms26199653

**Published:** 2025-10-03

**Authors:** Vincent Tambay, Valérie-Ann Raymond, Simon Turcotte, Marc Bilodeau

**Affiliations:** 1 Laboratoire d’hépatologie Cellulaire, Centre de Recherche du Centre Hospitalier de l’Université de Montréal, Montréal, QC H2X 0A9, Canada; 2 Biobanque et Base de Données Hépatobiliaire et Pancréatique, Centre hospitalier de l’Université de Montréal, Montréal, QC H2X 0A9, Canada; 3 Département de Chirurgie, Service de Transplantation Hépatique et de Chirurgie Hépatobiliaire et Pancréatique, Centre Hospitalier de l’Université de Montréal, Montréal, QC H2X 0A9, Canada; 4 Département de Médecine, Université de Montréal, Montréal, QC H3C 3J7, Canada

**Keywords:** liver, metabolism, hepatocellular carcinoma, metabolic reprogramming, glutamine, CB-839

## Abstract

Hepatocellular carcinoma (HCC) is the most prevalent primary liver cancer, with a poor prognosis due to late diagnosis, limited curative therapies, and underlying liver disease. Glutamine metabolism, a crucial pathway in cancer, remains poorly understood in HCC, which develops in an already metabolically dynamic organ. This study aimed to characterize glutamine metabolism in HCC. Glutamine metabolism in HCC was explored through in vitro analysis of neoplastic characteristics, experimental hepatocarcinogenesis in C57BL/6 mice, and examination of liver samples from patients with HCC, cirrhosis, and non-diseased liver. The evaluation included metabolite abundance and mRNA/protein expressions. In mice, tumors exhibited hyperactive glutaminolysis compared to adjacent tissue. Notably, glutaminase expression shifted from the liver isoform (GLS2) in normal and cirrhotic livers to the kidney isoform (GLS1) in HCC. In samples from patients, HCC tumors showed overexpression of glutamine synthetase and GLS1 along with a loss of GLS2 expression, providing excellent discrimination of HCC lesions from cirrhotic and normal liver samples. Inhibiting GLS1 with CB-839 significantly impacted glutamine metabolism in HCC cells while showing limited activity on normal hepatocytes. HCC tumors show reprogramming of GLS2 to GLS1, with a concomitant increase in glutamine synthetase. These characteristics can discriminate HCC from cirrhotic and normal liver tissues. Overexpressed GLS1 and loss of GLS2 within tumors convey an unfavorable prognosis in patients with HCC. Pharmacological inhibition of GLS1 in HCC cells successfully harnesses glutamine metabolism, representing an attractive target for novel therapeutic approaches.

## 1. Introduction

Hepatocellular carcinoma (HCC), arising from the neoplastic transformation of hepatocytes, is the most prevalent primary liver malignancy. It accounts for approximately 90% of all primary liver cancers, and current projections suggest that the annual global incidence will reach 1 million individuals worldwide in 2025 [1]. While the risk of HCC development has traditionally been linked to underlying cirrhosis caused by chronic viral and alcoholic liver diseases, the escalating prevalence of metabolic-associated steatotic liver diseases (MASLD) is anticipated to impose a substantial disease burden in North America and Europe [1,2]. Notably, HCC ranks as the 3rd most fatal cancer globally, with a median 5-year survival of approximately 20%, and thus represents one of the cancer types with the most pressing unmet needs [2,3].

Given that HCC occurs within the liver, a pivotal organ for systemic metabolism, the phenomenon of metabolic reprogramming during liver disease and hepatocarcinogenesis has become a focal point of investigation. The typical disease continuum leading to HCC involves chronic liver insult resulting in increased extracellular matrix deposition, progression of fibrosis into cirrhosis, and a heightened risk of HCC emergence [4,5,6]. Approximately 80–90% of HCC cases develop in the context of underlying cirrhosis [1,5]. Given the important role of metabolism in normal liver and that the occurrence of HCC is a multi-step process, perturbations of cell metabolism are bound to result from and contribute to hepatocarcinogenesis. From a metabolomic perspective, studies have shown distinct metabolic profiles in normal healthy liver, cirrhotic liver, and HCC [7,8,9,10,11,12]. Metabolic reprogramming is now acknowledged as a hallmark of cancer [13]. Tumors exhibit intrinsic and extrinsic factors influencing metabolic alterations, including tissue of origin, genetic mutations, altered cell signaling pathways, and the tumor microenvironment [14,15]. Investigating altered metabolism in HCC cells is crucial for a comprehensive understanding of its pathophysiology.

**Figure 1:**
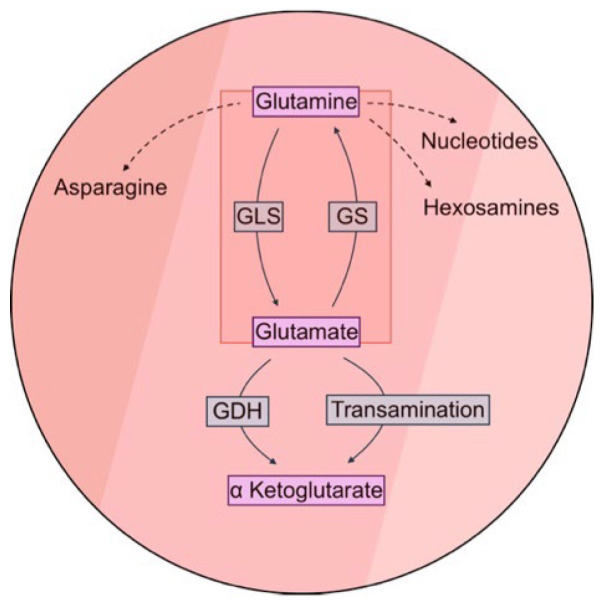
Intracellular glutamine-related metabolic pathways. Glutaminase (GLS) catalyzes glutaminolysis, whereas glutamine synthetase (GS) catalyzes glutaminogenesis. Glutamate is metabolized to α-ketoglutarate, a Krebs cycle intermediate, via glutamate dehydrogenase (GDH) or transamination. Dotted lines denote alternative pathways to glutaminolysis.

The non-essential amino acid glutamine holds immense importance in systemic and cell metabolism due to its versatility, including its contribution to the Krebs cycle, as well as the synthesis of non-essential amino acids, hexosamines, and nucleotides [16]. Glutamine breakdown is initiated through glutaminolysis, which is carried out by glutaminase (GLS), releasing glutamate and ammonia. GLS exists as two isoforms: (1) GLS1, whose expression is considered ubiquitous but most prominent in the kidney and brain, and (2) GLS2, the liver-type GLS [17]. Conversely, glutamine can be synthesized de novo from glutamate in an ATP-dependent fashion via glutamine synthetase (GS), a process known as glutaminogenesis [16] (Figure 1). The liver, a central hub for metabolism, orchestrates glutamine-related metabolism with organized zonation within lobules [18,19]. In periportal hepatocytes, GLS supports gluconeogenesis for euglycemic regulation and the urea cycle for ammonia detoxification [18,19]. Perivenous hepatocytes contribute to glutamine synthesis, sequestering non-metabolized ammonia as glutamine [18,19,20]. Despite being tightly regulated in normal liver, glutamine metabolism is frequently altered in various cancers [21].

In this study, we investigated the reprogramming of glutamine metabolism in HCC. We explored GLS and GS expression in murine HCC and in a single-center cohort of HCC patients from the Centre hospitalier de l’Université de Montréal (CHUM). Furthermore, we investigated the activity of glutaminolysis and glutaminogenesis in HCC compared to adjacent liver tissue. Finally, we assessed the diagnostic and prognostic implications, as well as the pathogenic relevance of glutamine metabolism reprogramming in HCC.

## 2. Results

### 2.1. Rewiring of Hepatic Glutamine Metabolism Correlates with Development and Progression of Murine Hepatocellular Carcinoma

**Figure 2:**
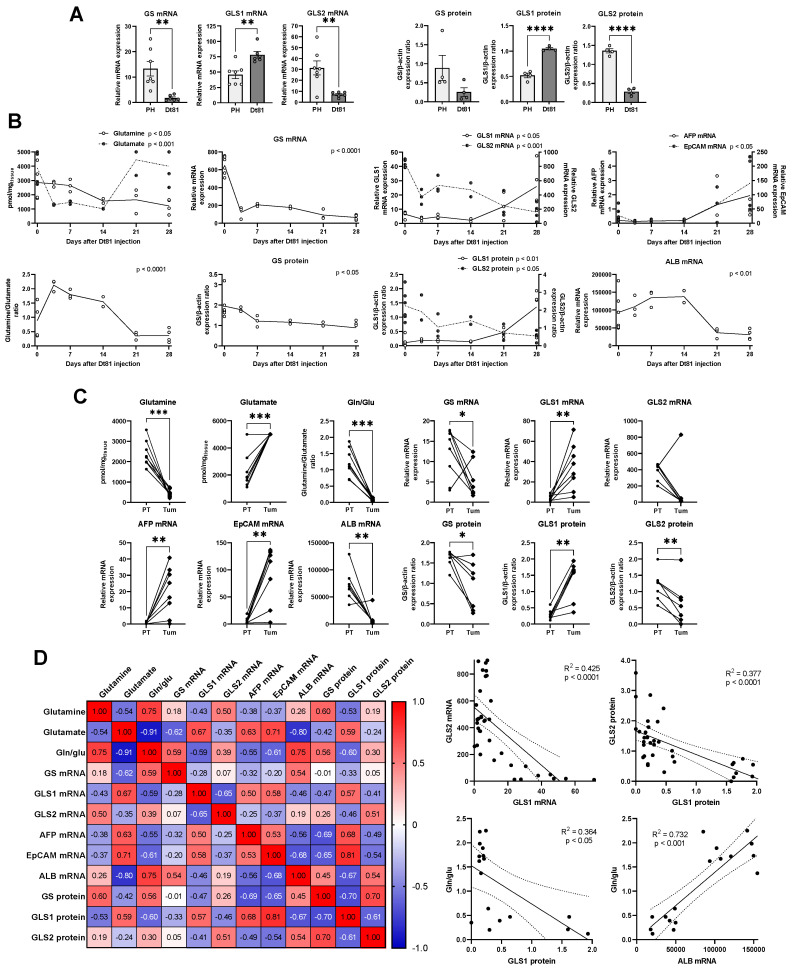
Remodeling of hepatic glutamine metabolism correlates with murine hepatocellular carcinoma tumorigenesis and progression. (**A**). Relative mRNA and protein expression of GS, GLS1), and GLS2 in primary murine hepatocytes (PH) and Dt81Hepa1-6 (Dt81) HCC cells. Data were compared using unpaired *t*-test. (**B**). HCC development in C57BL/6 mice sacrificed at 3.5, 7, 14, 21, or 28 days. Whole-liver glutamine, glutamate, GS, GLS1, GLS2, alpha-fetoprotein (AFP), epithelial cell adhesion molecule (EpCAM), and albumin (ALB) were measured. Data were compared using one-way ANOVA with Tukey’s post-test. (**C**). HCC induced in C57BL/6 mice; livers extracted 21 days after injection and separated into paired tumoral (Tum) and peritumoral (PT) samples. Data were compared for each liver using paired *t*-tests. (**D**). Pearson correlation coefficients for all parameters across all murine livers. Coefficients are represented in a correlation heatmap and chosen regressions as scatter plots. *: *p* < 0.05; **: *p* < 0.01; ***: *p* < 0.001; ****: *p* < 0.0001.

We first measured mRNA and protein levels of the 3 key enzymes involved in glutamine metabolism: GS, GLS1, and GLS2. A comparative analysis between our murine HCC cell line and cultured normal primary mouse hepatocytes isolated from the same strain revealed higher GS mRNA levels in normal hepatocytes compared to the HCC cell line. GLS1 mRNA and protein levels were higher in the Dt81 HCC cell line while GLS2 levels were higher in normal hepatocytes at both mRNA and protein levels. These findings suggested reprogramming of GLS in HCC. We then studied murine HCC in vivo to simulate different steps of the hepatocarcinogenic process. Our analysis revealed a time-dependent decrease in whole-liver glutamine (*p* < 0.05), and an increase in glutamate levels (*p* < 0.001) (Figure 2B). Consequently, the glutamine/glutamate ratio decreased significantly during tumor development (*p* < 0.0001). Hepatic GS and GLS2 mRNA decreased over time (both *p* < 0.0001), whereas GLS1 mRNA and protein levels increased (*p* < 0.05); GS and GLS2 also increased but only at the protein level (GS, *p* < 0.05; GLS2, *p* < 0.01). The expression of epithelial cell adhesion molecule (EpCAM), a marker of cancer cell stemness, also increased over time (*p* < 0.05), whereas albumin (ALB) mRNA decreased (*p* < 0.01).

These results led us to explore the specific changes in glutamine metabolism occurring within HCC tumors by separating murine livers into paired tumoral and adjacent peritumoral samples at the time of sacrifice (Figure 2C). Glutamine was decreased and glutamate increased specifically in HCC tissues (both *p* < 0.001); in accordance, the glutamine/glutamate ratio was decreased in tumors (*p* < 0.001). GS was decreased in tumors (*p* < 0.05), whereas GLS1 increased (*p* < 0.01). GLS2 protein levels were significantly decreased in tumors (*p* < 0.01). As expected, AFP and EpCAM were only found in tumors whereas ALB was much greater in adjacent tissues (all *p* < 0.01).

Finally, Pearson correlation analyses were performed for all measured parameters (Figure 2D). In liver tissues, glutamine and glutamate were inversely correlated (R = −0.54, *p* < 0.001), similar to GLS1 and the glutamine/glutamate ratio (*p* < 0.05). We also identified a negative correlation between the glutamine/glutamate ratio and AFP (*p* < 0.05) and EpCAM (*p* < 0.01). GLS1 and GLS2 correlated inversely with each other (both *p* < 0.0001). GLS1 positively correlated with AFP and EpCAM, but inversely with ALB. GLS2 protein expression positively correlated with ALB. These results emphasize the relevance of glutamine metabolism alterations during hepatocarcinogenesis within the context of metabolic reprogramming in HCC.

### 2.2. Discriminative Performance of Glutamine Metabolism Reprogramming in Human HCC Compared to Cirrhotic and Normal Livers

**Figure 3:**
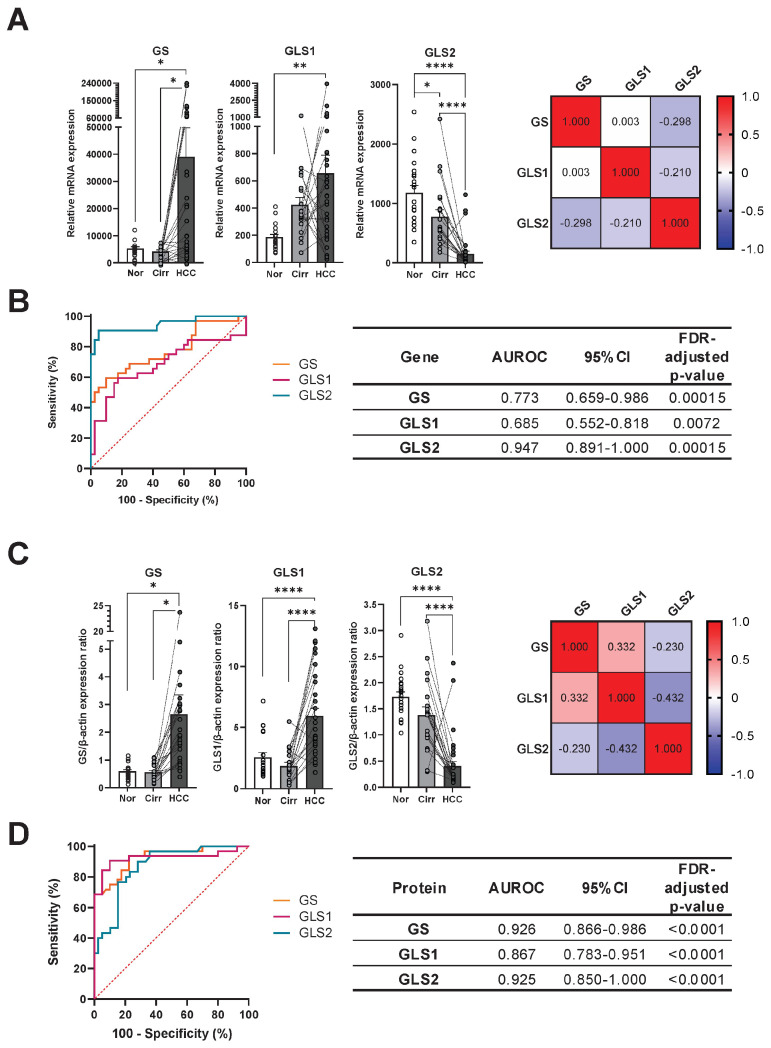
Discriminative performance of glutamine metabolism reprogramming in human HCC compared to cirrhotic and normal liver. (**A**). Tissue relative mRNA expression of GS, GLS1, and GLS2. Data were compared using one-way ANOVA with Tukey’s post-test. Correlation heatmap of Pearson correlation coefficients between GS, GLS1, and GLS2 for all samples. (**B**). ROC analyses for each gene between HCC samples and non-tumoral liver samples (cirrhotic and normal). (**C**). Tissue protein expression of GS, GLS1, and GLS2. Data were compared using one-way ANOVA with Tukey’s post-test, and Pearson correlation coefficients reported in a heatmap. (**D**). ROC analyses for each studied protein between HCC samples and non-tumoral liver samples (cirrhotic and normal). Paired cirrhotic and HCC samples are linked by dotted lines. AUROC: Area under ROC curve; 95%CI: 95% confidence interval; Nor: Normal liver (N = 20); Cirr: Cirrhotic liver (N = 20); HCC: Hepatocellular carcinoma tumor (N = 32). *: *p* < 0.05; **: *p* < 0.01; ****: *p* < 0.0001.

The established framework provided by the reprogramming of glutamine metabolism in our murine model served as a solid foundation for studying disruptions in glutamine metabolic pathways in human HCC. Our objective was to characterize glutamine metabolic reprogramming in patients with HCC, comparing tumoral tissues with underlying cirrhotic tissue from cancer patients and healthy liver tissues from individuals without primary liver disease (Figure 3). Leveraging our patient biobank, we identified samples from HCC, cirrhotic, and control liver tissues (Table 1).

We first examined the mRNA expression of genes involved in glutamine metabolism in HCC lesions compared to cirrhotic and control tissues (Figure 3A). GS was upregulated in HCC tissues compared to cirrhotic and control liver samples (both *p* < 0.05). Similarly, GLS1 expression was higher in HCC samples compared to normal tissues (*p* < 0.01). GLS2 expression was highest in normal tissue, intermediate in cirrhotic tissue (*p* < 0.05), and notably low in tumors (both *p* < 0.0001).

Next, ROC analyses were performed to ascertain the discriminative performance of glutamine metabolism reprogramming at the mRNA level between HCC tissues and non-tumoral (cirrhotic and control) liver tissues (Figure 3B). mRNA levels of individual genes were initially used as predictors of HCC or non-tumoral groups in all samples. GS and GLS1 exhibited fair performance, whereas that of GLS2 showed excellent ability to discriminate HCC lesions (AUROC = 0.947, *p* = 0.00015, 95% CI 0.891–1.000). Using tissue GLS2 expression, tumors could be identified with 90.63% sensitivity and 95.00% specificity using a cutoff value of <291.8.

To validate the findings observed for HCC at the mRNA level, GS, GLS1, and GLS2 proteins were evaluated (Figure 3C). GS protein levels were significantly lower in normal and cirrhotic tissues compared to HCC (both *p* < 0.05), similarly to GLS1 (both *p* < 0.0001). GLS2 was significantly lower in HCC tissues compared to normal or cirrhotic livers (both *p* < 0.0001). Additionally, Pearson correlation revealed a negative correlation between GLS1 and GLS2 (R = −0.43, *p* < 0.001, Figure 3C).

ROC analyses were then performed to identify their discriminative performance of these proteins (Figure 3D). GS protein levels showed excellent results, similarly to GLS2 (AUROC = 0.925, *p* < 0.0001, 95% CI GS: 0.866–0.986, GLS2: 0.850–1.000), followed by GLS1 protein levels, which also performed well but to a lesser degree. Considering GLS2 protein expression using a predefined cutoff (>0.85), samples were successfully classified as HCC with 90.63% sensitivity and 90.00% specificity.

### 2.3. Alterations in the Expression of Tumoral Glutaminase and Glutamine Synthetase Expression Bear Significant Prognostic Implications in Patients with Hepatocellular Carcinoma

**Figure 4:**
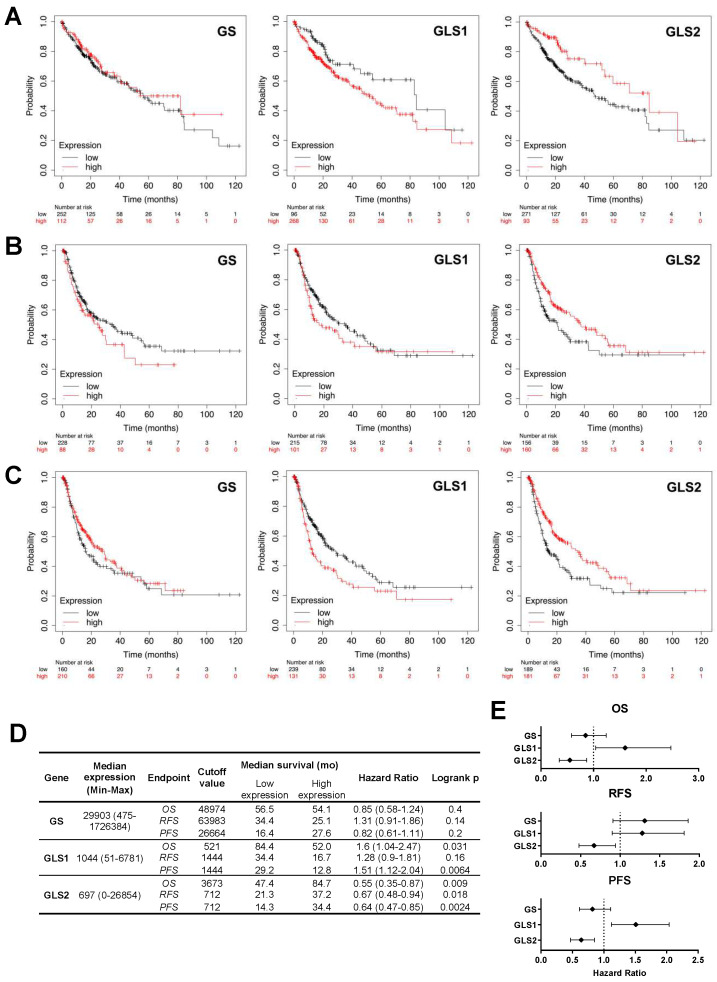
Prognostic significance of altered glutaminases and glutamine synthetase in HCC tumors. Survival analysis of The Cancer Genome Atlas Liver—Hepatocellular carcinoma (TCGA-LIHC) cohort using Kaplan–Meier plots. The TCGA-LIHC cohort was divided into low- or high-expressing tumors for GS, GLS1, or GLS2 genes, according to median expression. Median survival was compared using the log-rank test between low (black) and high (red) intratumoral expression cohorts for each gene, with (**A**). Overall survival (OS), (**B**). Relapse-free survival (RFS), and (**C**). Progression-free survival (PFS) as clinical endpoints. (**D**). Kaplan–Meier estimates and cohort stratification according to intratumoral gene expression. (**E**). Forest plots of hazard ratios from log-rank analysis for each dataset.

Given the robust performance of the reprogramming of glutamine metabolism-related genes and proteins in the ability to differentiate between HCC and normal or cirrhotic livers, we next focused on the prognostic value of glutamine-related metabolic alterations in patients with HCC. Consequently, we studied the predictive potential of GS, GLS1, and GLS2 in determining tumor aggressiveness and patient mortality as assessed through overall survival (OS), relapse-free survival (RFS), and progression-free survival (PFS) (Figure 4).

The influence of intratumoral GS, GLS1, and GLS2 mRNA expression on patient OS was first evaluated (Figure 4A). High expression of GLS1 was associated with a negative impact on prognosis, leading to a median OS of 49.6 months compared to 70.5 months in the low-expressing group, though not reaching statistical significance (HR = 1.31 (0.92–1.85)). Conversely, low tumoral expression of GLS2 was associated with a poorer OS of 45.7 months, compared to 61.7 months in patients with high-expressing tumors (HR = 0.72 (0.51–1.01). Next, relapse was used as the clinical endpoint to compare prognosis among HCC patients (Figure 4B). Individuals with low-GS or high-GLS1-expressing tumors experienced relapse more rapidly by approximately 4 months, though this did not attain statistical significance. Similarly, low tumor expression of GLS2 was associated with a 16-month decrease median in time to relapse (HR = 0.70 (0.50–0.97)). Finally, progression served as the third clinical endpoint (Figure 4C). Patients whose HCC tumors showed GLS1 overexpression tended to progress more rapidly (HR = 1.26 (0.94–1.68)), similar to those showing low GLS2 (*p* < 0.05, HR = 0.65 (0.49–0.88)). Summary data used for Kaplan–Meier analyses and comparative forest plots are presented in Figure 4D,E.

### 2.4. Pharmacological Targeting of Kidney Glutaminase Inhibits Glutaminolysis and Downstream Metabolism in Hepatocellular Carcinoma Cells

**Figure 5:**
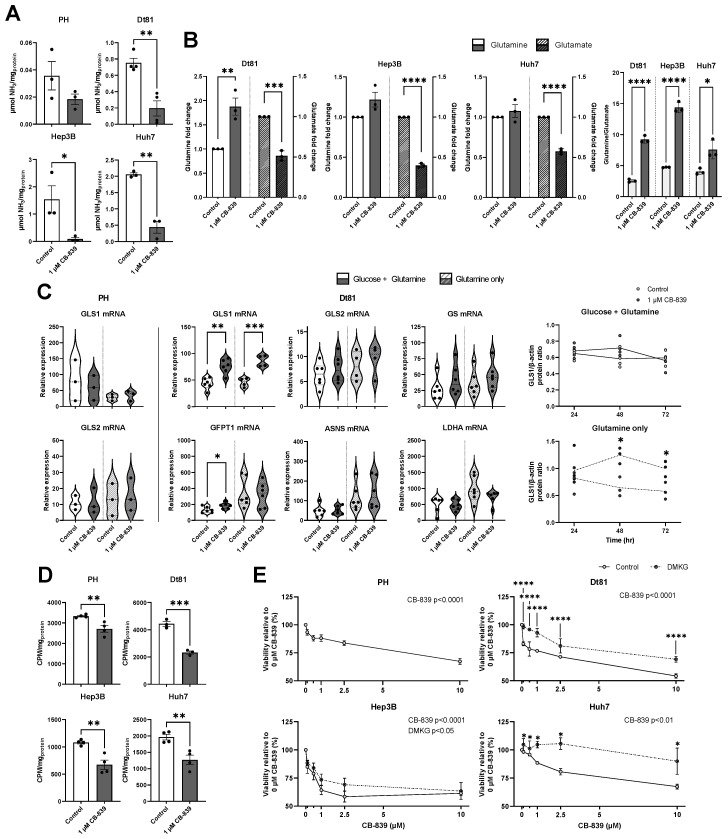
Pharmacological targeting of kidney glutaminase successfully inhibits glutaminolysis and glutamine-related metabolism in hepatocellular carcinoma cells. (**A**). Total ammonia production of cultured normal primary hepatocytes (PH), murine Dt81Hepa1-6 (Dt81) HCC cells, and human HCC Hep3B and Huh7 cells, as treated with or without 1 µM CB-839 (GLS1 inhibitor) for 24 h. Data were compared using unpaired *t*-test. (**B**). Intracellular glutamine and glutamate levels (fold change relative to control) and calculated ratios. Data were compared using unpaired *t*-tests. (**C**). Violin plots of relative mRNA expression of GLS1, GLS2, GS, GFPT1, ASNS, and LDHA at 24 h. Protein expression of GLS1 after exposure to CB-839. Data were compared using unpaired *t*-tests for mRNA and proteins (comparisons within each timepoint). (**D**). Cellular uptake of ^3^H-glutamine after CB-839 treatment (24 h). Statistical analysis was performed through unpaired *t*-tests. (**E**). Cell viability relative to 0 µM CB-839 in glucose/glutamine-rich media (control) or supplemented with DMKG. Cells were cultured in glucose/glutamine-rich media, unless stated otherwise. Statistics were performed using two-way ANOVA (factors: CB-839, medium): stars represent statistical differences between the control and DMKG groups for each CB-839 dose whereas the dose-dependent impact of CB-839 on cell viability reported as ‘p’. CPM: Counts per minute. *: *p* < 0.05; **: *p* < 0.01; ***: *p* < 0.001; ****: *p* < 0.0001.

Our comprehensive study of the expression and activity of glutaminolysis and glutaminogenesis highlighted GLS1 as a pivotal metabolic intermediate in HCC, unveiling a potential novel target for cancer therapy focusing on cell metabolism. We investigated the therapeutic potential of the selective GLS1 inhibitor CB-839 on glutamine-related metabolism and HCC cell survival. Initially, we examined the ability of CB-839 to inhibit glutamine breakdown in HCC cells by measuring the cellular production of ammonia (Figure 5A). CB-839 decreased ammonia production in Dt81 (*p* < 0.01), Hep3B (*p* < 0.05), and Huh7 (*p* < 0.01) HCC cells. CB-839 also significantly increased the glutamine/glutamate ratio in all HCC cells (Dt81 and Hep3B: *p* < 0.0001; Huh7: *p* < 0.05, Figure 5B).

To identify possible metabolic adaptations under CB-839 exposure, we measured the expression of various glutamine-related genes in murine HCC cells compared to normal hepatocytes (Figure 5C). Only in Dt81 cells, GLS1 mRNA was specifically increased by CB-839, in both glucose-deprived (*p* < 0.01) and glucose-replete (*p* < 0.001) conditions. Glucosamine-fructose-6-phosphate aminotransferase (GFPT1) mRNA also increased with CB-839 in glucose-replete conditions (*p* < 0.05). Moreover, GLS1 protein expression, in the absence of glucose, increased significantly in Dt81 cells under CB-839 treatment at 48 and 72 h (Figure 5C, both *p* < 0.05). ^3^H-glutamine uptake assays (Figure 5D) revealed that CB-839 decreased glutamine consumption in normal hepatocytes (*p* < 0.01), but to a much lesser extent than in Dt81 (*p* < 0.001). Likewise, CB-839 decreased glutamine consumption in Hep3B and Huh7 cells (both *p* < 0.01).

Finally, the cytotoxic effect of GLS1-dependent inhibition by CB-839 on HCC cells was evaluated using the MTT assay (Figure 5E). CB-839 significantly decreased cell viability dose-dependently in normal hepatocytes and Dt81 cells, (both *p* < 0.0001). However, the extent to which cell viability was decreased was lower in hepatocytes than in Dt81, especially at doses inferior to 2.5 µM. In Dt81, treatment with 10 µM CB-839 decreased viability by 46.0 ± 1.9% (*p* < 0.0001). Human HCC cell viability also decreased with CB-839 dose-dependently (Hep3B: *p* < 0.0001, Huh7: *p* < 0.01). Furthermore, when CB-839-exposed cells were treated with dimethyl-α-ketoglutarate (DMKG), a membrane-permeable ester of α-ketoglutarate, a metabolite downstream of glutaminolysis, cell viability was restored at all studied CB-839 doses (all *p* < 0.0001). Huh7 cells were much less sensitive to CB-839 in the presence of DMKG than in control conditions (all *p* < 0.05). Hep3B cells remained sensitive to CB-839 despite DMKG, though its presence did significantly improve cell survival (*p* < 0.05). Thus, CB-839-treated cells repleted with DMKG show partial restoration of cell viability, highlighting the importance of the glutamate-to-α-ketoglutarate pathway for HCC cell survival downstream of GLS1.

## 3. Discussion

In recent years, there has been growing interest in understanding the intricate metabolic reprogramming underlying liver diseases, particularly HCC. Given the pivotal role of the liver in systemic metabolism and the versatile function of glutamine in maintaining cellular integrity and promoting proliferation, our focus centered on unraveling the dynamic of glutaminolysis and glutaminogenesis in the context of HCC. Through comprehensive analyses of whole liver extracts of our immunocompetent murine HCC model, we highlighted a compelling narrative of HCC initiation and progression marked by a significant increase in glutamate levels and a decline in glutamine. The glutamine/glutamate ratio, serving as a quantitative indicator of the balance between glutamine catabolism and synthesis, exhibited a pronounced decrease over the course of HCC development. These changes strongly hinted at an overactivity of glutaminolysis relative to glutaminogenesis, suggesting a shift towards increased glutamine breakdown in the tumoral microenvironment.

Consequently, the fluctuations in glutamine and glutamate levels within HCC tumors prompted the formulation of the hypothesis that GS and GLS undergo simultaneous alterations during hepatocarcinogenesis. Decreased GS expression was a hallmark of our murine HCC model, whereas in human HCC lesions, GS expression was increased compared to cirrhotic and non-diseased liver. This discrepancy may arise from the highly controlled nature of our HCC model in contrast to the intricate, multi-factorial, and highly heterogeneous hepatocarcinogenic process observed in patients. Among the important signaling pathways governing GS expression is β-catenin [18,19]. Increased β-catenin signaling, primarily induced in HCC by activating mutations of CTNNB1 or loss-of-function mutations of AXIN1/2, can serve as an upregulator of GS, aligning with observations we made in our patient cohort [22]. Consistent with our findings, diffuse overexpression of GS in HCC has been evaluated as a potential diagnostic biomarker [23,24]. Furthermore, in our in vitro and in vivo murine HCC models, we observed significant shifts in GLS1 and GLS2 mRNA and protein levels, with GLS1 increasing and GLS2 decreasing specifically within tumoral tissues. Seeking to extend the translational relevance of these observations, we examined our patient cohort, revealing a clear reprogramming of the studied enzymes, GS, GLS1, and GLS2, in HCC lesions.

Moreover, we observed a negative correlation between GLS1 and GLS2 in our murine HCC model, mirroring our findings in human liver samples. This implies that the reduction in GLS2 observed in HCC is concurrent with an elevation in GLS1. This shift may appear intriguing in the context of HCC, considering that the normal liver already plays an important role in glutamine metabolism. However, the reprogramming of GLS could confer metabolic advantages to HCC cells, which often exhibit heightened demands for energy metabolites and biosynthetic precursors compared to non-tumoral hepatocytes. Given that GLS1 boasts higher affinity and faster catalytic activity for glutamine breakdown than GLS2, utilizing glutamine to meet the metabolic needs of cancer cells is likely to be optimized under GLS1 [25,26]. The elevated GLS activity of GLS1 in HCC should enable tumors to efficiently harness all available glutamine within the tumor microenvironment, thus supporting their metabolic requirements more effectively.

Furthermore, GLS2, as a target of p53, has been identified as a tumor suppressor, capable of inducing ferroptosis in HCC [27,28,29,30]. The diminished expression of GLS2 in HCC may be attributed to p53 mutations, which are known to occur in approximately 31% of tumors of this type [31]. Dedifferentiation during cancer initiation and progression could also contribute to the loss of GLS2, a marker for hepatocellular differentiation owing to its association with ALB. Additionally, GLS2 promoter hypermethylation has been proposed to contribute to its suppression in HCC [30,32]. Such mechanisms may collectively account for the under-expression of GLS2 observed in HCC, although remain to be further demonstrated. In contrast, GLS1 has been shown to act under the influence of oncogenes such as MYC and is linked to tumor invasion, metastasis, and heightened tumor aggressiveness in various malignancies [28,33,34].

In our murine model, we also observed a negative correlation between GLS1 and the glutamine/glutamate ratio, suggesting that upregulation of GLS1 is associated with increased glutaminolysis. Conversely, GLS2 exhibited a negative correlation with the glutamine/glutamate ratio, reinforcing this hypothesis that accelerated glutaminolysis in HCC is predominantly influenced by GLS1. AFP and EpCAM were linked to increased glutaminolysis and correlated with GLS1, suggesting that GLS1 may be a putative metabolic gene of HCC. Interestingly, previous studies have associated GLS1 expression with serum AFP levels in HCC [35,36].

Altogether, the altered expression of GS and reprogramming of GLS from GLS2 to GLS1 in HCC seem to be a promising avenue to ascertain the diagnosis of HCC. To explore this potential, we conducted ROC analyses using gene and protein expressions as input predictors to classify liver samples as either HCC or non-tumoral (cirrhotic and control) liver tissues. At the mRNA level, the tissue expression of GLS2 exhibited the greatest ability to classify tissues as tumors. The comprehensive characterization of glutamine metabolism reprogramming, encompassing GS and GLS1 overexpression and most importantly GLS2 loss, shows promising diagnostic significance for HCC. Though these findings are compelling, bootstrapped confidence intervals, cross-validation, or validation were not performed in an independent cohort, which represents a limitation of the current work. Future studies in larger and independent patient populations will be required to validate our exploratory findings, ideally including cross-validation strategies and multivariable modeling approaches to better confirm the diagnostic value of GS, GLS1, and GLS2 expression in HCC.

Beyond its ability to identify HCC tumor tissues, we assessed the prognostic value of glutamine metabolism reprogramming in terms of patient survival and cancer progression. Comparative analyses of intratumoral GS, GLS1, and GLS2 mRNA levels showed that intratumoral overexpression of GLS1 associated with higher patient mortality rates and shorter median intervals to progression. The fundamental role of GLS1 in explaining the increased glutaminolysis observed in HCC indicates its association with tumor aggressiveness, suggesting that GLS1-dependent glutaminolysis may be a metabolic cornerstone in HCC progression, though increasing effect size or optimizing cutoff values in such analyses may reveal if such associations are statistically significant. Conversely, high tumor expression of GLS2 was associated with decreased tumor aggressiveness, with patients exhibiting significantly longer OS, RFS, and PFS than those with low intratumoral expression. Since GLS2 is associated with hepatic differentiation, the loss of its expression in HCC may associate tumors with better dedifferentiation, and hence less aggressive. Such findings underscore the switch from GLS2 to GLS1 as a clinically relevant marker of HCC progression and prognostic stratification. However, a limitation of this study is that potential confounders, including etiology and vascular invasion, which may inherently influence patient survival, were not accounted for in patient stratification.

Furthermore, the distinctive transition of GLS expression to GLS1 in HCC suggests its pivotal role in glutamine metabolism. To study this hypothesis, we used the GLS1 inhibitor, CB-839. When HCC cells were exposed to CB-839, we observed a significant inhibition of ammonia production and increased the glutamine/glutamate ratio. These data again suggest that GLS1 serves as the primary source of glutaminolytic activity in HCC cells, and that targeting GLS1 could selectively inhibit glutaminolysis in HCC. We showed that CB-839 altered GLS1 expression in HCC cells, but not in primary hepatocytes. Specifically, CB-839 increased GLS1 mRNA and protein levels, potentially arising from transcriptional and translational adaptations in order to compensate for the blockade its activity. We also showed that CB-839 is powerful enough to inhibit glutamine metabolism in HCC cells to a degree that renders glutamine less usable, as its uptake was decreased under treatment. Finally, GLS1 inhibition induced dose-dependent cell death of all tested HCC cell lines. Normal hepatocytes also displayed sensitivity to CB-839 treatment, suggesting that the overlapping GLS1 activity in such cells cannot be ignored. However, it should be noted that the metabolism of cultured primary hepatocytes may not always precisely reflect in situ liver conditions [37]. Nevertheless, the effect of GLS1 inhibition on hepatocyte survival remained much less pronounced than on HCC cells. Replenishing cells with a downstream metabolite of GLS1, DMKG, restored viability, implying that glutamine metabolized through GLS1 is critical in supporting mitochondrial metabolism by fueling carbons to the TCA cycle. Interestingly, Hep3B remained sensitive to CB-839 treatment despite the presence of DMKG, suggesting that certain HCC cells rely on GLS1 for alternate metabolic pathways. Overall, targeting GLS activity holds potential in future treatment approaches for HCC. In the clinical setting, CB-839 (Telaglenastat) has been investigated for the treatment of various malignancies (Table 2); HCC has yet to be included in such trials. Despite promising therapeutic potential in combination therapies targeting glutamine metabolism [38], few preclinical studies have sought to elucidate the role of GLS1 inhibition in HCC. Based on the present findings, further investigations should focus on targeting glutaminolysis to mitigate HCC aggressiveness and progression.

## 4. Materials and Methods

### 4.1. Cell Lines, Hepatocyte Isolation, and Culture Reagents

Authenticated human Hep3B and Huh7 HCC cells were purchased from the American Type Culture Collection (Manassas, VA, USA). Highly tumorigenic murine Dt81Hepa1-6 cells (Dt81) were derived through in vivo passage of parental Hepa1-6 cells in C57BL/6 mice [39]. Primary hepatocytes (PH) were isolated from male C57BL/6 mice (20–22 g) purchased from Charles River (Saint-Constant, QC, Canada) using the two-step perfusion method [40]. Cells were cultured in Dulbecco’s Modified Eagle Medium (DMEM) at 37 °C and 5% CO_2_.

Reagents, including DMEM high glucose and glutamine/glucose/pyruvate-free media, Fetal Bovine Serum, Penicillin/Streptomycin, Leibovitz’s L-15 medium, D-glucose, and L-glutamine, were purchased from Invitrogen (Burlington, ON, Canada). Type IV collagenase was obtained from Worthington Biochemical (Lakewood, NJ, USA). CB-839 was purchased from Cayman Chemical (Ann Arbor, MI, USA). All other products were from Sigma-Aldrich (Oakville, ON, Canada) unless stated otherwise.

### 4.2. Animals and Experimental Hepatocarcinogenesis

Male C57BL/6 mice (20–22 g) were fed ad libitum with a Chow diet. HCC was induced using Dt81Hepa1–6 cells as previously described [39]. At sacrifice, livers were harvested, either whole or after dissection between tumoral and adjacent peritumoral specimens. Mice without surgical intervention served as controls. All animal procedures were approved by the “Comité institutionnel de protection animale du CHUM” and respected guidelines from the Canadian Council on Animal Care (approval #IP20014MBs and #S11043MBs).

### 4.3. Biobank Samples and Patient Cohort

Patients were recruited for participation within the “Banque de données cliniques et biologiques et d’échantillons biologiques associés à des fins de recherche sur les cancers hépatobiliaires et pancréatiques” of the Centre hospitalier de l’Université de Montréal, Montréal, QC, Canada (approval #09.237). Prior to surgery, written informed consent was obtained from each patient. This research protocol was conducted in accordance with the Declaration of Helsinki and was approved by the “Comité d’éthique de la recherche du Centre de recherche du CHUM”. A total of 52 participants (controls, *n* = 20; HCC, *n* = 32) were included in this study. The principal inclusion criterion for the controls and HCC patients was eligibility for liver resection, while the exclusion criteria for the controls included the presence of hepatitis or fibrosis. Adjacent cirrhotic liver samples were obtained from 20 of the 32 HCC participants.

### 4.4. Gene Expression Analysis

RNA was isolated by phenol–chloroform extraction with TRIzol^®^ (Invitrogen (Burlington, ON, Canada)) reagent following the manufacturer’s instructions. mRNA extracts (250 ng) were reverse transcribed into cDNA with the QuantiTect Reverse Transcription Kit (QIAGEN (Toronto, ON, Canada)). Real-time PCR was performed using the QuantiTect SYBR Green PCR Kit (QIAGEN (Toronto, ON, Canada) in a Real Time Rotor-Gene 3000 Thermocycler (Corbett Research, Sydney, Australia). Relative mRNA expression was calculated using the delta-delta CT method with 3 reference genes for murine samples (PPIA, HPRT1, and H2AFZ) and 2 reference genes for human samples (S9 and HMBS) [41].

### 4.5. Western Blotting

Tissue and cell samples were lysed and homogenized in RIPA buffer with protease/phosphatase inhibitors, and then sonicated and quantified using the Bradford assay. Proteins (50 µg) were boiled prior to electrophoresis in a 10% SDS-polyacrylamide gel and then transferred to PVDF membranes. Membranes were blocked in PBST with 10% skim milk for one hour and washed. Proteins were probed with anti-GS (1:5000 (11037-2-AP, Proteintech, Rosemont, IL, USA)), anti-GLS1 (1:1000 (19958-1-AP, Proteintech, Rosemont, IL, USA)), and anti-GLS2 (1:1000 (PA5-21113, Invitrogen, Burlington, ON, Canada)) for two hours in PBST containing 1% milk. Membranes were washed and incubated with HRP-conjugated anti-rabbit IgG (1:5000 (AB_3952131, BD Biosciences, San Diego, CA, USA)) in PBST containing 5% milk for two hours. β-actin was probed using anti-Actin kit (1:10,000, Calbiochem (Darmstadt, Germany)) in 1% milk for two hours, washed, and followed by HRP-conjugated anti-mouse IgM (1:20,000, Calbiochem (Darmstadt, Germany)) in 5% milk for one hour. All incubations were performed at 21 °C. Membranes were washed prior to chemiluminescence revelation using the Novex^TM^ ECL Chemiluminescent Substrate Reagent Kit (Invitrogen (Burlington, ON, Canada)) according to the manufacturer’s protocol. Membrane washes consisted of 3 × 10 min at room temperature in PBST.

### 4.6. Glutamine and Glutamate Quantification

Liver tissue metabolites were quantified by liquid chromatography/mass spectrometry (LC-MS) as previously described [7]. For the quantification of cellular glutamine and glutamate, metabolites were extracted using 75% acetonitrile, 1% formic acid, 20 µM d_5_-glutamine (C/D/N Isotopes (Pointe-Claire, QC, Canada)), and 20 µM d_5_-glutamate (TRC (Toronto, ON, Canada)) as internal standards. Samples were sonicated using a Q700 sonicator (Qsonica (Newtown, CT, USA)). Homogenates were centrifuged (20,000× *g*, 15 min, 4 °C), and supernatants were cleaned using Phree 1mL tubes (Phenomenex (Torrance, CA, USA)). Filtrates were analyzed in duplicate by LC-MS separation (Nexera X2; Shimadzu (Japan)) performed using a Poroshell 120 HILIC-Z, 2.1 × 100 mm, 2.7 µm particle size HPLC column preceded by a 2.1 × 5 mm guard column at 30 °C at a flow of 0.8 mL/min using the gradient: A, 20 mM ammonium formate in water (pH 3.0); B, 20 mM ammonium formate in acetonitrile:water (9:1, pH 3.0); 10 min 100% B; 11 min 70% B; 5 min 100% B. Positive ion electrospray ionization detection (QTRAP 6500, SCIEX) was performed.

### 4.7. Assessment of Cell Viability

Cell viability was assessed by a 3-h incubation with (3-(4,5-Dimethylthiazol-2-yl)-2,5-diphenyltetrazolium bromide) tetrazolium salt (MTT) from Bioshop (Burlington, ON, Canada). Viability was measured by optical density (540 nm) measured in a BioTek Instruments spectrophotometer (Winooski, VT, USA).

### 4.8. H-Glutamine Uptake Assay

Cells were incubated with glutamine/glucose-free medium containing 20 nM L-[3,4-^3^H(N)]-Glutamine [1 µCi/mL] purchased from Perkin Elmer (Woodbridge, ON, Canada) for one hour. ^3^H-glutamine was removed from the cell medium followed by three washes with 10% trichloroacetic acid (4 °C). Monolayers were dried with 100% methanol, lysed [0.5 M NaOH, 1 mM EDTA, 0.1% Triton X-100], and exposed to Ultima Gold liquid scintillation cocktail (Perkin Elmer (Woodbridge, ON, Canada)). Liquid scintillation counting was performed using a Tri-Carb 2800TR analyzer (Perkin Elmer (Woodbridge, ON, Canada)).

### 4.9. Ammonia Measurement

Ammonia was measured in cell culture media and cultured cells. Media were diluted 1:20 in triethanolamine [pH 8.6] prior to measurement. Cells were washed with PBS and lysed by sonication in triethanolamine [pH 8.6]. Ammonia was measured using a Cobas c111 analyzer (Roche (Laval, QC, Canada)). Ammonia was quantified using a Randox AM1015 kit (Crumlin, UK).

### 4.10. Survival Analysis of HCC Patients

Survival analyses were performed using the KM-plotter database (accessed 16 september 2025) [42]. With RNAseq data from The Cancer Genome Atlas—Liver Hepatocellular Carinoma (TCGA-LIHC) program, comparative survival analyses of HCC patients (*n* = 364) were performed between low and high intratumoral mRNA expression cohorts for GS, GLS1, and GLS2 [42]. The TCGA-LIHC cohort was divided into low and high-expressing tumor cohorts for each gene according to median expression as the cutoff value between lower and upper halves of mRNA expression as a function of *p* values and hazard ratio values. No patient stratification was performed. The prognostic value of intratumoral mRNA expression levels was measured through Kaplan–Meier plotting with “death”, “relapse”, or “progression” as clinical endpoints. Median survival and comparative hazard ratios were calculated for each dataset.

### 4.11. Statistical Analysis

All data were collected and compared from at least three independent experiments, animals, or tissue samples using Prism 10.6.1 (Boston, MA, USA). Unless stated otherwise, data are represented as mean ± standard error. Various statistical approaches were used to compare data, namely unpaired *t*-test, paired *t*-test, one-way ANOVA with Tukey’s post-test for multiple comparisons, two-way ANOVA with Dunn-Sidak’s correction for multiple comparisons, receiver operating characteristic analysis, Pearson correlation, as well as log-rank comparison for survival analyses. For patient studies, multiple comparisons for ANOVA, ROC and Kaplan–Meier analyses were corrected for FDR using the Benjamini–Hochberg procedure.

## 5. Conclusions

The reprogramming of glutamine metabolism emerges as a defining feature of HCC, with distinctive alterations of glutamine breakdown and synthesis pathways, exemplified by the transition from GLS2 to GLS. By promoting GLS1 expression, HCC tumors capitalize on their inherent nutrient-seeking behavior, heightening their capacity to metabolize glutamine and thereby foster tumor growth and cancer progression. Our comprehensive approach, encompassing the evaluation of GS, GLS1, and GLS2, underscores the potential of glutamine-related reprogramming as a tool for the diagnosis of HCC in a normal or cirrhotic environment Moreover, GLS reprogramming may be associated with HCC aggressiveness as demonstrated by its impact on patients’ prognostic. Finally, as the pharmacological inhibition of GLS1 with CB-839 impacts HCC cell survival, this paves the way to use this target in clinical trials of patients with HCC.

## Figures and Tables

**Table 1:** Clinicopathological data of HCC patients and control participants in this study.

		HCC (*n* = 32)	Cirrhosis (*n* = 20)	Control (*n* = 20)
Demographics	Male	26 (81.3%)	18 (90.0%)	14 (70.0%)
	Median age (mean), years	68 (63.8)	68 (63.3)	60 (60.5)
Cirrhosis		25 (78.1%)	20 (100.0%)	0 (0.0%)
Etiology	Alcohol	1 (3.1%)	1 (5.0%)	-
	HBV	5 (15.6%)	4 (20.0%)	-
	HCV	4 (12.5%)	4 (20.0%)	-
	MASLD	6 (18.8%)	6 (30.0%)	-
	Other/Unknown	16 (50%)	5 (25.0%)	-
Tumor biology	Solitary, *n* (%)	12 (71.9%)	-	-
	Greatest tumor dimension, mean (cm)	7.1	-	-
Vascular invasion	Microvascular	8 (25.0%)	-	-
	Macrovascular	8 (25.0%)	-	-
	None	14 (43.8%)	-	-
	Unknown	4 (12.5%)	-	-
Grade	I (*n*, %)	3 (9.4%)	-	-
	II (*n*, %)	17 (53.1%)	-	-
	III (*n*, %)	6 (18.8%)	-	-
	IV (*n*, %)	2 (6.3%)	-	-
	Unknown (*n*, %)	4 (12.5%)	-	-
TNM staging	T1	12 (37.5%)	-	-
	T2	12 (37.5%)	-	-
	T3	5 (15.6%)	-	-
	T4	1 (3.1%)	-	-
	TX	2 (6.3%)	-	-
	N0	4 (12.5%)	-	-
	N1	1 (3.1%)	-	-
	NX	27 (84.4%)	-	-
	MX	32 (100%)	-	-

**Table 2:** Clinical trials assessing anti-neoplastic efficacy and/or pharmacological safety of GLS1 inhibitor Telaglenastat (CB-839).

NCT #	Title	Status	Conditions	Intervention	Sponsor (Collaborators)	Phase
04250545	Testing of the Anti Cancer Drugs CB-839 HCl (Telaglenastat) and MLN0128 (Sapanisertib) in Advanced Stage Non-small Cell Lung Cancer	Active, not recruiting	Leptomeningeal neoplasm, non-small cell lung cancer, metastatic malignant brain neoplasm	Telaglenastat HCl, sapanisertib	NCI	I/Ib
03965845	A Study of Telaglenastat (CB-839) in Combination With Palbociclib in Patients With Solid Tumors	Completed	Non-small cell lung cancer, colorectal carcinoma	Telaglenastat, palbociclib	Calithera Biosciences, Inc.	Ib/II
04824937	Telaglenastat + Talazoparib In Prostate Cancer	Unknown (12 September 2025)	Metastatic prostate cancer	Telaglenastat, talazoparib	Massachusetts General Hospital (Calithera Biosciences, Inc.; Pfizer; Prostate Cancer Foundation)	II
03875313	Study of CB-839 (Telaglenastat) in Combination With Talazoparib in Patients With Solid Tumors	Terminated	Renal cell carcinoma, triple-negative breast cancer, colorectal cancer	Telaglenastat, talazoparib	Calithera Biosciences, Inc.	I/II
03528642	Telaglenastat With Radiation Therapy and Temozolomide in Treating Patients With IDH-Mutated Diffuse Astrocytoma or Anaplastic Astrocytoma	Active, not recruiting	Astrocytoma	Telaglenastat, temozolomide, radiation therapy	NCI	I
03872427	Testing Whether Cancers With Specific Mutations Respond Better to Glutaminase Inhibitor, Telaglenastat Hydrochloride, Anti-Cancer Treatment, BeGIN Study	Active, not recruiting	Advanced/unresectable/metastatic malignant solid neoplasm, NF1-mutant malignant peripheral nerve sheath tumor	Telaglenastat HCl	NCI	II
03163667	CB-839 With Everolimus vs. Placebo With Everolimus in Participants With Renal Cell Carcinoma (RCC) (ENTRATA)	Completed	Clear-cell renal cell carcinoma	Telaglenastat everolimus	Calithera Biosciences, Inc.	II
04265534	KEAPSAKE: A Study of Telaglenastat (CB-839) With Standard-of-Care Chemoimmunotherapy in 1L KEAP1/NRF2-Mutated, Nonsquamous NSCLC (KEAPSAKE)	Terminated	Non-small cell lung cancer	Telaglenastat, carboplatin, pemetrexed, pembrolizumab	Calithera Biosciences, Inc.	II
03831932	Telaglenastat Hydrochloride and Osimertinib in Treating Patients With EGFR-Mutated Stage IV Non-small Cell Lung Cancer	Active, not recruiting	Advanced/metastatic non-small cell lung cancer	Telaglenastat HCl, osimertinib	NCI	I/II
02771626	Study CB-839 in Combination With Nivolumab in Patients With Melanoma, Clear Cell Renal Cell Carcinoma (ccRCC) and Non-Small Cell Lung Cancer (NSCLC)	Terminated	Clear-cell renal cell carcinoma, melanoma, non-small cell lung cancer	Telaglenastat, nivolumab	Calithera Biosciences, Inc.	I/II
03428217	CANTATA: CB-839 With Cabozantinib vs. Cabozantinib With Placebo in Patients With Metastatic Renal Cell Carcinoma (CANTATA)	Completed	Advanced/metastatic renal cell carcinoma	Telaglenastat, cabozantinib	Calithera Biosciences, Inc.	II
04540965	Impact of a Histamine H2 Receptor Antagonist (H2RA) on the Pharmacokinetics (PK) of Telaglenastat in Healthy Subjects	Completed	Drug interaction	Telaglenastat, famotidine	Calithera Biosciences, Inc. (Novotech (Australia) Pty Limited)	I
03057600	Study of CB-839 in Combination w/Paclitaxel in Participants of African Ancestry and Non-African Ancestry With Advanced Triple Negative Breast Cancer (TNBC)	Completed	Triple-negative breast cancer	Telaglenastat, paclitaxel	Calithera Biosciences, Inc.	II
03263429	Novel PET/CT Imaging Biomarkers of CB-839 in Combination With Panitumumab and Irinotecan in Patients With Metastatic and Refractory RAS Wildtype Colorectal Cancer	Completed	Colorectal cancer	Telaglenastat, panitumumab, irinotecan	Vanderbilt-Ingram Cancer Center (NCI, Calithera Biosciences, Inc.)	I/II
03798678	CB-839 HCl in Combination With Carfilzomib and Dexamethasone in Treating Patients With Recurrent or Refractory Multiple Myeloma	Active, not recruiting	Recurrent multiple myeloma	Telaglenastat, dexamethasone, carfilzomib	NCI	I
02071888	Study of the Glutaminase Inhibitor CB-839 in Hematological Tumors	Completed	Non-Hodgkin’s lymphoma	Telaglenastat, dexamethasone, pomalidomide	Calithera Biosciences, Inc.	I
02071927	Study of the Glutaminase Inhibitor CB-839 in Leukemia	Completed	Acute myeloid leukemia, acute lymphoid leukemia	Telaglenastat, azacitidine	Calithera Biosciences, Inc.	I
02861300	CB-839 + Capecitabine in Solid Tumors and Fluoropyrimidine Resistant PIK3CA Mutant Colorectal Cancer	Completed	Colorectal cancer	Telaglenastat, capecitabine	David Bajor, Case Comprehensive Cancer Center	I/II
02071862	Study of Glutaminase Inhibitor CB-839 in Solid Tumors	Completed	Triple-negative breast cancer, non-small cell lung cancer, renal cell carcinoma, mesothelioma, gastrointestinal stromal tumors	Telaglenastat, paclitaxel, everolimus, erlotnib, docetaxel, cabozantinib	Calithera Biosciences, Inc.	I
03047993	Glutaminase Inhibitor CB-839 and Azacitidine in Treating Patients with Advanced Myelodysplastic Syndrome	Completed	Acute myeloid leukemia, chronic myeloid leukemia	Telaglenastat, azacitidine	M. D. Anderson Cancer Center	I/II
03944902	CB-839 in Combination with Niraparib in Platinum-Resistant BRCA-Wild-type Ovarian Cancer	Terminated	Ovarian cancer	Telaglenastat, Niraparib	University of Alabama at Birmingham	I

## Data Availability

All data generated or analyzed during this study are included in this published article. Any additional inquiries may be directed to the corresponding author.

## Abbreviations

The following abbreviations are used in this manuscript:

AFP	Alpha-fetoprotein
ALB	Albumin
ANOVA	Analysis of variance
ASNS	Asparagine synthetase
AUROC	Area under receiver operating characteristic curve
CHUM	Centre hospitalier de l’Université de Montréal
DMEM	Dulbecco’s modified essential medium
DMKG	Dimethyl-α-ketoglutarate
Dt81	Dt81Hepa1-6
EpCAM	Epithelial cell adhesion molecule
GFPT1	Glucosamine-fructose-6-phosphate aminotransferase
GLS	Glutaminase
GLS1	Kidney glutaminase
GLS2	Liver glutaminase
GS	Glutamine synthetase
HMBS	Hydroxymethylbilane synthase
HPRT1	Hypoxanthine phosphoribosyltransferase 1
HRP	Horseradish peroxidase
H2AFZ	Histone H2A.Z variant
LC-MS	Liquid chromatography/mass spectrometry
LDHA	Lactate dehydrogenase
MTT	(3-(4,5-Dimethylthiazol-2-yl)-2,5-diphenyltetrazolium bromide) tetrazolium salt
OS	Overall survival
PFS	Progression-free survival
PH	Primary hepatocyte
PPIA	Peptidylprolyl isomerase A
RFS	Relapse-free survival
ROC	Receiver operating characteristic
S9	40S ribosomal protein S9
TCGA	The Cancer Genome Atlas
TGCA-LIHC	The Cancer Genome Atlas—Liver Hepatocellular Carcinoma
